# Adoption Factors of the Electronic Health Record: A Systematic Review

**DOI:** 10.2196/medinform.5525

**Published:** 2016-06-01

**Authors:** Clemens Scott Kruse, Krysta Kothman, Keshia Anerobi, Lillian Abanaka

**Affiliations:** ^1^ Texas State University School of Health Administration San Marcos, TX United States

**Keywords:** electronic health record, information technology, HITECH Act, health information technology

## Abstract

**Background:**

The Health Information Technology for Economic and Clinical Health (HITECH) was a significant piece of legislation in America that served as a catalyst for the adoption of health information technology. Following implementation of the HITECH Act, Health Information Technology (HIT) experienced broad adoption of Electronic Health Records (EHR), despite skepticism exhibited by many providers for the transition to an electronic system. A thorough review of EHR adoption facilitator and barriers provides ongoing support for the continuation of EHR implementation across various health care structures, possibly leading to a reduction in associated economic expenditures.

**Objective:**

The purpose of this review is to compile a current and comprehensive list of facilitators and barriers to the adoption of the EHR in the United States.

**Methods:**

Authors searched Cumulative Index of Nursing and Allied Health Literature (CINAHL) and MEDLINE, 01/01/2012–09/01/2015, core clinical/academic journals, MEDLINE full text, and evaluated only articles germane to our research objective. Team members selected a final list of articles through consensus meetings (n=31). Multiple research team members thoroughly read each article to confirm applicability and study conclusions, thereby increasing validity.

**Results:**

Group members identified common facilitators and barriers associated with the EHR adoption process. In total, 25 adoption facilitators were identified in the literature occurring 109 times; the majority of which were efficiency, hospital size, quality, access to data, perceived value, and ability to transfer information. A total of 23 barriers to adoption were identified in the literature, appearing 95 times; the majority of which were cost, time consuming, perception of uselessness, transition of data, facility location, and implementation issues.

**Conclusions:**

The 25 facilitators and 23 barriers to the adoption of the EHR continue to reveal a preoccupation on cost, despite incentives in the HITECH Act. Limited financial backing and outdated technology were also common barriers frequently mentioned during data review. Future public policy should include incentives commensurate with those in the HITECH Act to maintain strong adoption rates.

## Introduction

### Background

Currently in the United States, expenditures associated with health care average 17.5% of the gross domestic product (GDP) [1]. The Health Information Technology for Economic and Clinical Health (HITECH) Act was initiated in 2009 and, as described by Samuel (2014), implemented goals of “widespread” adoption of Electronic Health Records (EHRs) that should realize nationwide savings in the health care industry [2]. Although much research exists in support of the policy makers’ agenda tied to the HITECH Act, the widespread adoption process leaves many providers reluctant to move forward due to concerns of financial pressures, technology limitations, and potential unintended errors related to limited knowledge of the EHR [3]. There is plenty of literature that supports the idea that adoption of Health Information Technology (HIT), specifically the EHR, presents great potential value to the health care industry in our nation [3]. Through the implementation of HIT, patients, providers, and intermediaries can expect “efficiency, effectiveness, and safety of health care” [4]. The potential for great savings, efficiency, and quality through the adoption of the EHR created high expectations from the federal government, and President Bush even expected ubiquitous adoption by the year 2014 [5]. However, only 55% of nationwide providers had fulfilled the HITECH Act requests by the end of 2014 [5]. With financial-savings estimates ranging from $77-$371 billion throughout the country following broad implementation, adoption of the EHR is essential for all who are involved [6]. A thorough review of EHR adoption facilitator and barrier factors provides ongoing support for the continuation of EHR implementation across various health care structures, possibly leading to a reduction in associated economic expenditures. Several researchers have examined adoption factors and barriers, but a gap in the literature exists that places these factors into an affinity diagram to identify those facilitators and barriers to adoption most often cited [7].

### Objective

The purpose of this review is to compile a current and comprehensive list of facilitators and barriers to the adoption of the EHR in the United States, and create an affinity diagram that orders these items by frequency of occurrence. Although frequency of occurrence in the literature does not necessarily identify the most important factors, it may help policy makers prioritize levels of effort for maximum effectiveness and the results of this review should enable future studies to explore the significance and order of importance.

## Methods

### Search

We searched for research on the topic of both facilitators and barriers to adoption of the EHR. A quick look at the Medical Subject Heading (MeSH) in PubMed terms shows no clear association with the term “adoption” in the sense of “selection”. As a result, a combination of Boolean operators and several similar terms were employed in a manner that would be likely to capture of the desired articles. Additionally, two terms are closely associated with the electronic records: the electronic health record, and the electronic medical record (EMR). While these terms are distinct in the HIT field, they are often used interchangeably throughout the literature, so both were included in the search terms. We also accepted studies and reviews on the topic, but only if they were published in academic journals or indexed in MEDLINE.

### Data

Articles for this review were gathered from two separate databases: Cumulative Index of Nursing and Allied Health Literature (CINAHL) Academic Search Complete via Ebson B Stephens Company (EBSCO Host), and PubMed (MEDLINE Complete). Search criteria were not limited to any specific focus. Instead, we searched for EHR or EMR adoption factors and barriers to adoption in patient care facilities in general. An iterative, nonlinear search string was created through PubMed and a similar string was used with Boolean operators in CINAHL.

**Figure 1:**
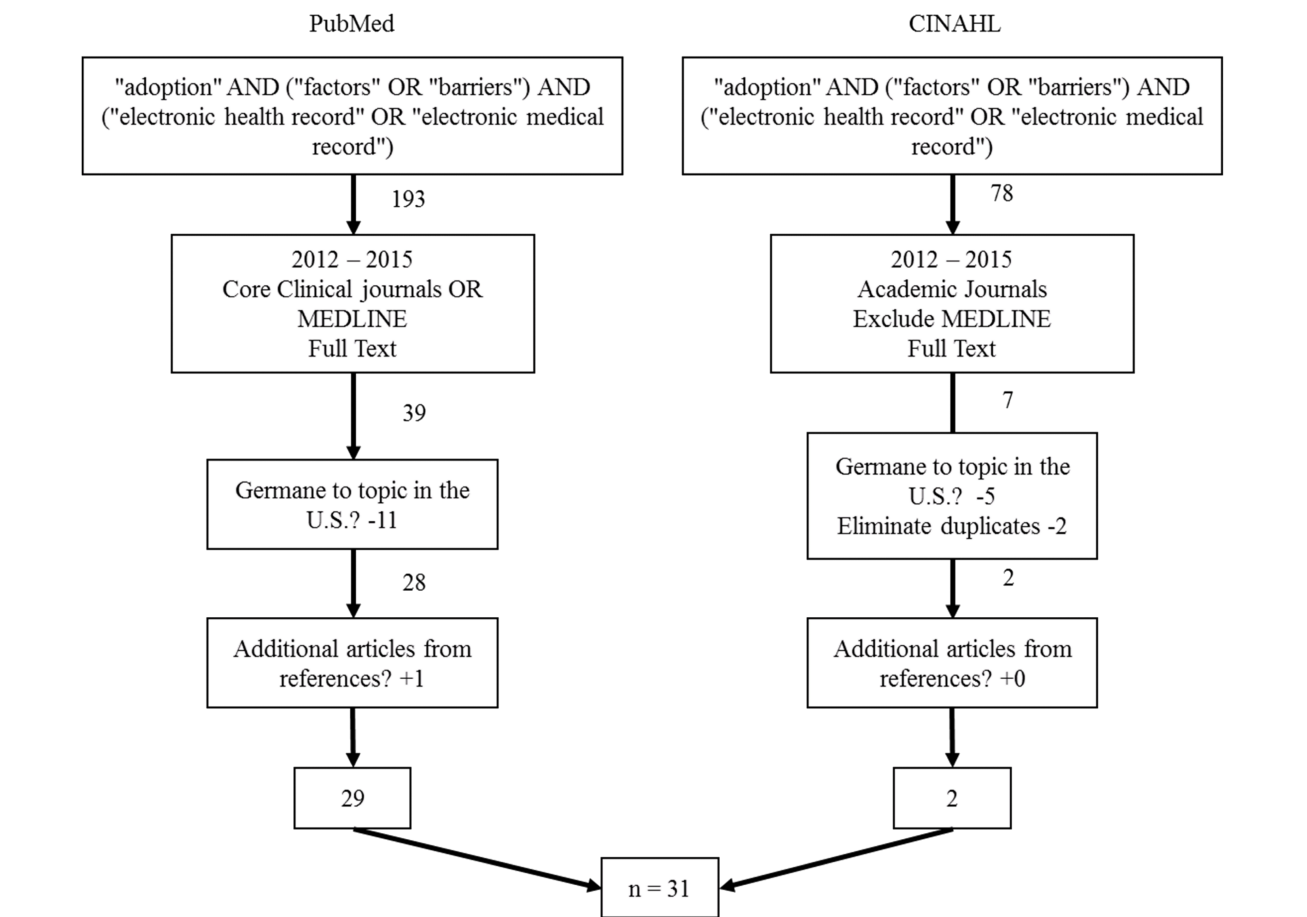
The search process with inclusion and exclusion criteria.

Figure 1 illustrates the search process, with the associated inclusion and exclusion criteria. As depicted, we narrowed the focus of the review to 1/1/2012–9/1/2015, core clinical/academic journals, full text. From this process, 60 articles were identified. The beginning of 2012 was chosen because it is one year after incentives for Meaningful Use incentives became available. The entire process of article selection is illustrated in Figure 1 (Literature review process). Authors agreed ahead of time on acceptable criteria for articles included in the review in an effort to increase the inter-rater reliability.

Using the criteria agreed upon, we independently read abstracts of these articles to determine if the research was germane to our topic, then we discussed our findings to reach consensus. Once consensus was reached, we examined the references in the remaining 30 articles to identify additional research that was not captured with our search string; one additional article was identified for the sample through this process. The final sample included 31 articles. The inter-rater reliability for the initial selection of titles was very good (kappa=.789). Our group of five divided the articles into sets that overlapped. We met again to discuss the merits of these articles, and through this meeting, we identified common themes in the literature of both facilitators and barriers to adoption. Consensus was reached on all 31 articles (kappa=1.0, excellent).

We decided to include systematic reviews in the sample because the data in the reviews would help validate our review. A total of three reviews were included and integrated into a literature matrix with the other articles. The literature matrix consisted of date of publication, journal, authors, titles, study designs, data sources, and pertinent details on both facilitators and barriers to the adoption of the EHR. Studies and reviews were sorted by date of publication (newest to oldest), by author (alphabetical), and they were assigned numbers that correspond to those in the references. The numbers are not sequential in Table 1 because several of the articles were used in the background section, so their numbers are lower than the start of those called up in the review. From this matrix, multiple affinity diagrams were created that illustrate the frequency of facilitators, barriers, study designs, and sources of data.

## Results

### Summary of Findings

We identified 31 unique publications that addressed facilitators and/or barriers to adoption of the EHR. Our analysis identified 25 facilitators for and 23 barriers to adoption. A portion of our literature matrix is included in Table 1. Many factors that some studies listed as facilitators were listed by others as barriers.

**Table 1:** Summarized facilitators and barriers.

Authors	Facilitators	Barriers
Kruse CS, et al [8]	Access to information Error reduction Transfer of information Long-run cost savings Clinical and administrative efficiency Project planning Security Time savings Staff retention	Initial cost User perceptions Implementation problems External factors Training Cultural change Future upgrades Necessary maintenance
Cucciniello M, et al [9]	Commitment promotion Role defining System impacts assessments	Change processes
McCullough JM, et al [10]	Availability of clinical data Support from management Competition	Competition
Tang, et al [11]	Availability of RECs	none specified
Abramson EL, et al [12]	Size of hospital (bed size)	Cost Lack of incentive Lack of interoperability Competitiveness Ongoing cost of maintenance
Ben-Zion R et al [13]	Executive management support Alignment with firm strategy Economic competiveness Knowledge management Patient empowerment	Cost-benefit asymmetry Lack of standard protocols for data exchange Uncertainty over implementation cost User resistance Breaches in security Patient privacy
D'Amore JD, et al [14]	Continuity of care document	Omission or misuse of LOINC Excess precision in timestamps Omission or misuse of UCUM in meds Omission or misuse of RxNorm Omission or misuse of dose amount Omission or misuse of allergic reactions Omission or misuse of allergy severity Omission or misuse of dose frequency Omission of result interpretation Omission of result reference range
Jones EB, Furukawa MF [15]	Engage patients and family in their care Improve care coordination Improve population and public health Quality recognition	Health centers with large share of Hispanics and Blacks had lower adoption rates Centers located in rural areas Health center size, income status and region Health centers with larger share of patients whose family incomes were below poverty level had lower rate of EHR adoption
Kruse CS, et al [7]	Size of hospital (bed size) Competiveness Urban locations Users cognitive ability User attitude toward information Workflow impact Communication among users	Patients’ age Rural locations Computer anxiety
Samuel CA [2]	Patients enrolled in Medicare or Medicaid Metropolitan status Increased financial incentives	Health professional shortage areas Minority concentration
Sockolow PS, et al [16]	Increase in productivity Improved clinical notes Reduced time to reimbursement Improved communication among staff	Incomplete medication information Incomplete hospital-stay information
Ancker JS, et al [17]	Monetary incentives Efficiency (fewer providers needed) Efficiency (practice sites) Effectiveness (fewer patients) Practice size	Cost Lack of tech assistance
Audet AM, et al [18]	Size of practice Ability to search for patients by diagnosis Ability to list patients overdue for preventative care Sort patients by specific laboratory results	Cost lack of experience Lack of tech-support infrastructure
Baillie CA, et al [19]	Reduce readmission rates	Existing data may not serve well in a predictive model
Cheung SK, et al [20]	Efficiency Reduction of medical errors Ability to share patient information in public sector Eliminate need to store paper records Eliminate illegibility of practice partners	Patient unfriendliness Limited consultant time Cost concerns Computer use more time consuming Concerns on data migrations from paper to system Insufficient space for computer installation
Georgiou A, et al [21]	Laboratory order forms contained bar codes for easier ordering A unique bar code for patient details Unique bar codes for each test A test order episode barcode	EMR test order problems Handwritten request on an EMR order Order number problem Multiple forms EMR order incorrect Change of test Add-on test No information provided Longer data entry time
Hamid F, Cline TW [5]	EHR satisfaction increased when users understood the benefits Supportive management Training programs	Cost Perceived lack of usefulness and provider autonomy Time consuming
Iqbual U, et al [22]	Perceived usefulness Perceived ease to use Computer self-efficacy Security Intention to use	Clinics with high number of outpatient visits Subjective norm
Kirkendall ES, et al [23]	Communication Job satisfaction Quality and patient data Quality and safety of patient care Employee understanding and support Organizational support The “Rights” of patient care	Transition of data
Middleton B, et al [24]	Monetary incentives Improve effectiveness Improve efficiency	Increased training burden Alert fatigue
Patel V, et al [25]	Financial incentives Size of practice	Lack of interoperability standards
Shen X, et al [26]	Size of practice	Cost Lack of integration with other systems Lack of national guidelines for implementation
Xierali IM, et al [27]	Health maintenance organizations more likely to adopt EHR Those with faculty status more likely to adopt EHR	Medically underserved locations less likely to adopt EHR Geographic health professional shortage areas less likely to adopt EHR International medical graduates less likely to adopt EHR Group practice/solo practice and small practice physicians less likely to adopt EHR
Menachemi N, et al [28]	HMO penetration into market	Competition Low income patients
DesRoches CM, et al [29]	Size of facility Incentives	Cost Size of facility
Decker SL, et al [30]	Size of organization	Age
Hudson JS, et al [31]	Hospital setting Improved outcomes Reduce duplicative tests Integrate levels of care Improve communication Greater readability	Cost
Jamoom E, et al [32]	Age Size of practice Enhanced patient care	none specified
Leu MG, et al [33]	Size of practice	Cost Productivity Customizability (right fit)
Linder JA et al [34]	Better for structured documenters Better for free text documenters	Decrease in quality of care for dictator note takers
Ramaiah M, et al [35]	Workflow can be optimized Access to electronic information e-prescriptions	Workflow often ad-hoc in nature Check-backs of scripts still time consuming Medical literacy of clerks inhibits smooth scheduling Information must still be verified Lack of IT experience of staff Uncertainty of time Uncertainty of cost
Rea S, et al [36]	Secondary use of data Natural language processing	Privacy and security
Ronquillo JG [37]	Genome-associated care Reduce error More efficient care More effective care Control costs	Privacy and security
Wang T, Biederman S [38]	Reduce error Improve quality of care Deliver more effective care	Cost
Soares N, et al [39]	Improve clinician satisfaction Improve clinical efficiency Improve parent satisfaction	Cost Technical assistance Organizational barriers No consensus among peer organizations
Hacker K, et al [40]		Disruption of care Lack of interoperability Disruption of workflow Increased patient-cycle time Breakdown in communication Fragmentation of information Inflexible processes Physician overload

### Facilitators

As depicted in Table 1, various articles used similar, but not exact terms. While compiling the results into Table 2, several factors were similar enough to be combined. *User perception/perceived usefulness* [5,9,27,31], was combined with *user attitude toward information* [7,22,23,36]. Table 2 is organized to rank order each factor that serves as a facilitator for EHR adoption. The center column identifies the article in which the factor was observed–the numbers correspond to the number assigned in order of mention (Introduction), followed by the order analyzed (Table 1), and the numbers match those assigned to these articles in the references. The last column numbers the occurrences. There were a total of 25 facilitators, and they were found a total of 109 times in the literature.

From the facilitators listed, *efficiency, organization size, and improved quality* were listed 12%, 9%, and 9% of the total occurrences of all facilitators mentioned in the literature, respectively. *Access to patient care, user perception/perceived usefulness, ability to transfer information* and *incentives* were identified in the literature 7%, 6%, 6%, and 5%, respectively. *Error reduction, time savings*, and *competitiveness* were all listed 4% of all occurrences. The rest of the barriers were mentioned three or less times, so we grouped them into a category of miscellaneous.

### Barriers

As depicted in Table 1, various articles used similar, but not the exact terms. While compiling the results into Table 3, several barriers were similar enough to be combined. This occurred more often in the barrier table than the facilitator table. *Interoperability* was combined with *no standard protocol for data exchange* [12,22,26,40]. *Training* was combined with *maintenance and upgrades* [8,12,21,24]. The barrier of *Staff shortages* was combined with *overworked* [2,27,40]. *Privacy* was combined with *security* [10,36,37]. *Lack of infrastructure* was combined with *lack of space* [18,20]. Finally, *missing data* was combined with *omission of result, interpretation,* and *omission of result reference range* [14,16,21]. There were a total of 23 barriers, and they were found a total of 95 times in the literature.

**Table 2:** Facilitators identified in the literature.

Facilitators	Occurrences by article reference number	Total occurrences
Efficiency	2,7,8,15,16,17,19,20,23,25,29,31,33	13
Hospital size^a^	7,12,16,24,25,26,28,29,31,32	11
Improved quality	15,18,21,22,23,26,30,31,32,33	10
Access to patient data	8,10,15,19,20,22,28,29	8
User perception/perceived usefulness	5,7,9,21,22,26,30	7
Ability to transfer information	8,9,19,28,29,30	6
Communication	7,8,15,22,30	5
Executive management support	1,5,9,10,13	6
Incentives	2,16,21,23	5
Error reduction	8,19,31,32	4
Time savings	5,8,15,20	4
Competiveness^a^	7,10,13,27	4
Security	8,21,22	3
Improved population health	2,15,22	3
Continuity of care document	2,15,40	3
Urban/more developed locations/status^a^	2,7,26	3
Knowledge/IT management	11,13,15	3
Staff retention	8,16	2
Long run cost savings	8,31	2
Alignment with strategy	1,13	2
Project planning	8	1
Patient empowerment	1	1
Patient engagement	14	1
Effectiveness	32	1
Genome associated care	31	1

^a^Statistical association identified through retrospective studies, rather than answers to “why” in a survey or interview.

**Table 3:** Barriers identified in the literature.

Barriers	Occurrences by article reference number	Total occurrences
Cost	5,8,12,13,16,17,19,25,28,30,32, 33,34,37,38	16
Time consuming	5,19,20,32,34,39	6
User perception/perceived lack of usefulness	5,8,13,17,19,34	6
Transition of data	13,19,20,22,28,34	6
Facility location (rural areas)/characteristics^a^	2,7,14,21,28	6
Implementation issues	8,13,19,20,25	5
User/patient resistance	7,9,13,19,20	5
Lack of tech assistance/experience	13,16,29,33,38	5
Interoperability/no standard protocols for data exchange	12,21,25,39	4
Medical error	15,20,23,40	4
Training, maintenance, upgrades	8,12,20,23	4
Lack of agility to make changes	20,32,39	3
Staff shortages/overworked	2,26,39	3
Privacy and/or security	13,35,36	3
Missing data	15,20,40	3
External factors^a^	8,26,38	3
Competiveness	12,10,27	3
Provider or patient age^a^	7,29	2
Race & income disparities^a^	2,15	2
Lack of infrastructure and/or space for systems	17,19	2
Need organizational cultural change	8,38	2
Lack of incentives	12	1
IMGs less likely to adapt	26	1

^a^Statistical association identified through retrospective studies, rather than answers to “why” in a survey or interview.

The barrier most often identified in the literature was cost (17%, 16/95). This factor included the following: *initial cost, implementation cost, maintenance cost*, and *training cost*. The barriers of *too time consuming, user perception/perceived lack of usefulness, transition of data,* and *facility location* were each identified 6% of the time (6/95). *Implementation issues, user/patient resistance* and *lack of technical assistance or experience*, were listed 5% of all occurrences (5/95). *Lack of interoperability, medical error, training, maintenance, and upgrades* were all listed 4% of all occurrences (4/95). The rest of the barriers were mentioned three or less times, so we grouped them into a category of miscellaneous.

As depicted in Tables 2 and 3, two facilitating factors and four barriers to EHR adoption are followed by a superscript letter. These factors appeared in the literature, but they were identified through statistical associations by researchers conducting retrospective studies. We included these factors in the review because the retrospective studies add value overall, but they are set apart because they are factors that really cannot be easily changed; therefore, they do not offer administrators and policy makers much actionable information.

From the 31 articles included in the review, 3 (10%) were reviews, and 9 (29%) were mixed methods. The remaining articles were a combination of retrospective, observational, cross-sectional, or descriptive. Of the articles reviewed, 17 (55%) analyzed secondary data, 12 (39%) collected primary data, and 4 (13%) used a mixture of sources. Thirteen (42%) of the articles in the review collected primary data through a survey, interview, or combination of both.

## Discussion

### Principal Findings

We found it interesting how often perception plays into interviews and surveys, and in the case of this review, resulted in one or more factors appearing as both an enabler and a barrier, based on the perception of the interviewee. Error is one example of that phenomenon. It is listed as a facilitator (mentioned 4% of the time), *using the EHR to prevent error* [8,20,32,33] and as a barrier (mentioned 4% of the time), *use of the EHR can cause error* [14,16,21,24]. User perceptions were also listed on both sides for monetary factors: the cost-related facilitator was *incentives* (mentioned 5% of the time), and the cost-related barrier was *cost* (mentioned 17% of the time). One more dichotomy was time-related factors: the facilitator factor, *efficiency* (mentioned 12% of the time), and the barrier, *time consuming* (mentioned 6% of the time). Some interviewees listed *ability to transfer information* (6%) as a facilitator, while others listed *interoperability/no standard protocols for data exchange* (4%) as a barrier.

Results from this review are in line with others performed along the same lines. Cost is repeatedly a primary barrier to the adoption of the EHR [5,8,12,13,17,18,20,26, 28,31,33,34,35,38,39]. Several factors were reinforced by this review that highlight organizational characteristics such as size and location [7,8]. Location is a difficult barrier to overcome. It is not a mystery to anyone that rural communities often struggle to overcome barriers such as cost, bandwidth, and user/patient acceptance, a point supported by the literature [2,7,15,22,29]. Unfortunately, very few solutions are offered to this group; at a minimum policy should look to assist those who lag behind the rest of the adopters [29]. Small, rural communities are the slowest to adopt, and their size is a major disadvantage in terms of budget and technical agility. Policy should look to a range of factors to lever, such as organizational, cultural, technological, and financial considerations [9].

Many factors play a role in establishing an environment conducive to the adoption of the EHR. This review was not intended to establish causality, but instead, it was designed to identify the frequency with which facilitators and barriers are discussed in the literature. It is hoped that by this review, data-driven studies can be developed to strengthen the validity of the factors listed.

### Limitations

This paper provides a review of the factors associated with adoption of EHR systems. Interrater reliability was calculated for both the search terms and titles selected, as well as the consensus-building activity surrounding the final selection of the 31 articles. In that regard, reliability of the results are strong.

Validity was strengthened by these results aligning with those of previous reviews. This addresses internal validity, but external validity would be limited to the United States because articles that focused on other countries were excluded from the review. Another limitation is that EHR adoption and usage were often self-reported by physicians, and social-desirability bias may have led physicians to overestimate actual usage.

### Conclusion

Users and nonusers alike are concerned about similar topics such as efficiency, quality, and interoperability. This review supports the findings of other reviews. Additional research remains necessary to assess the EHR system adoption factors in health care organizations in future years. Within the constantly changing environment of health care in the United States, health care decision makers are gradually adopting the EHRs, but adoption is far from ubiquitous. Country-level advantages will likely not emerge until everyone adopts a fully interoperable EHR.

## Abbreviations

CINAHL: Cumulative Index of Nursing and Allied Health Literature
EBSCO Host: Ebson B Stephens Company
EHR: electronic health records
EMR: electronic medical records
GDP: gross domestic product
HITECH: The Health Information Technology for Economic and Clinical Health
MeSH: Medical subject headings from the American National Library of Medicine
